# New therapies for obesity

**DOI:** 10.1093/cvr/cvac176

**Published:** 2022-11-30

**Authors:** Dimitris Papamargaritis, Carel W le Roux, Jens J Holst, Melanie J Davies

**Affiliations:** Diabetes Research Centre, Leicester General Hospital, University of Leicester College of Medicine Biological Sciences and Psychology, Leicester LE5 4PW, UK; Diabetes Complications Research Centre, Conway Institute, University College Dublin, Dublin 4, Ireland; Diabetes Research Centre, Ulster University, Coleraine BT52 1SA, UK; Department of Biomedical Sciences and the NNF Center for Basic Metabolic Research, University of Copenhagen Panum Institute, Copenhagen 2200, Denmark; Diabetes Research Centre, Leicester General Hospital, University of Leicester College of Medicine Biological Sciences and Psychology, Leicester LE5 4PW, UK

**Keywords:** Obesity, Pharmacotherapy, Bariatric surgery, Semaglutide, Tirzepatide, Liraglutide

## Abstract

Obesity is a chronic disease associated with serious complications and increased mortality. Weight loss (WL) through lifestyle changes results in modest WL long-term possibly due to compensatory biological adaptations (increased appetite and reduced energy expenditure) promoting weight gain. Bariatric surgery was until recently the only intervention that consistently resulted in ≥ 15% WL and maintenance. Our better understanding of the endocrine regulation of appetite has led to the development of new medications over the last decade for the treatment of obesity with main target the reduction of appetite. The efficacy of semaglutide 2.4 mg/week—the latest glucagon-like peptide-1 (GLP-1) receptor analogue—on WL for people with obesity suggests that we are entering a new era in obesity pharmacotherapy where ≥15% WL is feasible. Moreover, the WL achieved with the dual agonist tirzepatide (GLP-1/glucose-dependent insulinotropic polypeptide) for people with type 2 diabetes and most recently also obesity, indicate that combining the GLP-1 with other gut hormones may lead to additional WL compared with GLP-1 receptor analogues alone and in the future, multi-agonist molecules may offer the potential to bridge further the efficacy gap between bariatric surgery and the currently available pharmacotherapies.

**This article is part of the Spotlight Issue on Obesity, Metabolism, and Diabetes.**

## 1. Introduction

Obesity is a complex, chronic, progressive, and relapsing disease characterized by abnormal or excessive body fat that impairs health.^1^ It is one of the greatest global public health challenges, considering that 13% of the global population lives with obesity and that its prevalence has been tripled since 1975.^2^ Obesity drives the pathogenesis of multiple metabolic and mechanical complications including type 2 diabetes (T2D), hypertension, dyslipidaemia, sleep apnoea, cardiovascular disease, non-alcoholic fatty liver disease, infertility, and osteoarthritis^3^ which result in a decreased life expectancy of 5–20 years and increased healthcare costs.^4–7^

Lifestyle interventions are the cornerstones for the management of obesity, but even the most intensive programmes still commonly only achieve 5–10% weight loss (WL) and long-term weight maintenance remains a challenge.^8^ Although 5–10% WL reduces cardiometabolic risk factors it may not be enough to make a difference to the lives of people with BMI ≥35 kg/m^2^ (Class II obesity and above) and/or to reverse some obesity-related complications such as sleep apnoea and T2D.^9,10^ Until recently, pharmacotherapy to achieve and maintain ≥10% WL along with suitable tolerability and safety remained an unmet challenge. Bariatric surgery was the only intervention that consistently resulted in ≥15% WL and weight maintenance long-term.^11^ This amount of WL led to improved quality of life (QoL), significant health benefits and reduced mortality.^11–13^ Despite the considerable benefits of bariatric surgery, it is not feasible or scalable as a population-wide intervention. Recent clinical trials with advanced therapeutic candidates including new glucagon-like peptide-1 receptor agonists (GLP-1 RA) and dual agonists demonstrate that the gap of sustained WL between bariatric surgery and pharmacotherapy is gradually closing.^14,15^

Here, we review the available interventions for WL and weight maintenance with a focus on pharmacological therapies for obesity approved over the last decade as well as the emerging development of new obesity pharmacotherapies. We also discuss the future directions of obesity pharmacotherapy and the research priorities to support implementation of these treatments in clinical practice.

## 2. Obesity treatments

### 2.1 Lifestyle interventions

Lifestyle interventions usually include support for health-improving behavioural changes including diet, increasing physical activity, and reducing sedentary time. Moderate intensity lifestyle interventions such as 500–600 kcal deficit diet together with advice to increase physical activity to 150 min/week usually lead to a WL of 2–5% at 12 months and weight maintenance remain a challenge.

Intensive lifestyle interventions, which often include partial or total meal replacements, intensive behavioural therapy (IBT), and/or structured exercise, can lead up to ≈10% WL at the end of the first year.^16,17^

In the DROPLET study, a community delivered, low energy (810 kcal/day) total diet replacement programme with formula products as the sole food during the first 8 weeks followed by food reintroduction resulted in mean WL of 10.7 kg at 12 months compared with 3.1 kg at the usual care group in people with obesity.^18^ However, at the 3-year follow-up, mean WL was 6.2 kg at the intervention arm compared with 2.7 kg at the usual care^19^ with 24% of participants achieving ≥10% WL in the total diet replacement programme compared with 13% in usual care, suggesting that for this smaller number of patients this treatment was effective.^19^

Structured exercise programmes can usually add 1.5–3.5 kg WL to a dietary intervention, and they have multiple other health benefits such as improvement in body composition, physical function, and cardiorespiratory fitness.^20^ There is limited evidence on the effect of structured exercise on weight maintenance after weight-loss through diet,^20^ but a recent clinical trial showed that the addition of a 52-week structured but flexible aerobic exercise programme after a low-calorie diet programme (mean WL 12%) can lead to 4.1 kg less weight gain compared with people who continue with their usual physical activity.^21^ However, long-term maintenance of the recommended amount of moderate to vigorous physical activity can be challenging.^22^

The largest trial assessing the effectiveness of intensive lifestyle interventions was the Look AHEAD study, where 5145 people with obesity and T2D were randomized to receive intensive lifestyle support (intervention group) vs. a structured education programme (usual care group).^23^ The intensive lifestyle group lost 8.6% of their initial body weight at 1 year and 4.7% at 8 years while the usual care arm lost 0.7% at 1 year and 2.1% at 8 years.^8^ In the intensive lifestyle group, 37.7% of study participants achieved ≥10% WL at 1-year post-operatively, and 39.3% of them (324/2144, 15.1% of the total population at the intensive lifestyle group) were able to maintain ≥10% WL for 8 years.^8^

Overall, the Look AHEAD intervention did not demonstrate a reduction in cardiovascular events or a reduction at the risk for new onset heart failure or atrial fibrillation.^24–26^ However, in a post-hoc analysis, those participants in the intervention arm who lost ≥10% of their baseline body weight during the first year (early good responders) had 20% lower risk for cardiovascular events over a 10 years follow-up period.^27^ Similarly, a 10% decrease in BMI over the first year in participants at the Look AHEAD study was associated with a 31% lower risk of incident heart failure, when a 10% decrease in BMI over a 4-year follow-up period was associated with a 20% lower risk of incident heart failure.^25^ These results suggest that ≥10% WL is associated with cardiovascular benefits for people with obesity and T2D. Moreover, the REVERSE-AF study also showed that ≥10% weight reduction results in 88% reversal from persistent to paroxysmal or no atrial fibrillation.^28^

In general, weight regain is common after lifestyle interventions and approximately 80% of weight lost is expected to be regained over the next 5 years.^29^ Only 10–25% of individuals who will undergo different intensity lifestyle interventions will be able to lose and maintain ≥10% WL long-term. The rest of individuals could be considered non-responders to the lifestyle intervention and will need further interventions to achieve and maintain significant WL.

#### 2.1.1 Why is it so challenging to maintain weight loss with lifestyle changes?

The weight regain after significant WL with lifestyle changes is not simply attributable to the loss of motivation or compliance from the patients. Instead, it is driven by potent biological mechanisms that stimulate food intake and reduce energy expenditure on the background of an ‘obesogenic’ environment, where ultra-processed, high-calorie foods are easily accessible, physical activity is reduced and sedentary time increased.^30,31^ The major drivers of weight regain after treatments that caused significant WL, include the persistence of a lower resting metabolic rate (RMR) (metabolic adaptation), the lower energy consumption during weight-bearing activities, and the persistence of increased appetite, probably mediated through long-lasting increased orexigenic and decreased anorexic signals.^30,31^ Overall, the increased appetite and the reduced energy expenditure during the weight-reduced state results in a feeling of constant and exhausting effort to maintain the achieved WL and to prevent the seemingly unavoidable weight regain over time.^30,32^

RMR is mainly determined by body composition and accounts for 60–70% of 24 h total energy expenditure in humans.^31,33^ However, RMR in response to WL is often reduced to a greater extent than would be expected based on the measured changes in body composition. This physiological mechanism is called ‘metabolic adaptation’ and is one of the reasons why the body resists further WL and individuals regain weight so easily.^33^ For example, the participants of the ‘Biggest Loser’ television program lost, on average, 40% of their body weight, and their mean RMR decreased by 610 kcal/day—this was on average 275 kcal/day lower compared with what was expected based on their body composition.^34^ This metabolic adaptation persisted 6 years later despite regaining two-thirds of the lost weight.^33,34^

**Figure 1:**
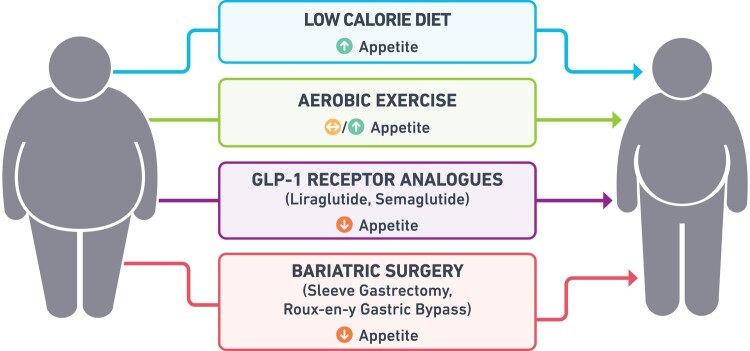
The effect of different weight loss interventions [low-calorie diet, exercise, pharmacotherapy (GLP-1 receptor analogues), and bariatric surgery] on appetite.

In addition, most people who manage to lose ≥10% of their body weight through low-calorie diet experience an increase in their appetite compared with baseline (*Figure 1*).^35^ Potential mediators of the increased appetite are the elevated levels of the hunger hormone ghrelin as well as the reduction in leptin and perhaps also satiety gut hormones such as peptide YY (PYY), amylin, and cholecystokinin.^35^ These changes remain even after 52 weeks from the completion of the low-calorie diet and despite that participants experience weight regain, for instance, 5.5 kg at the end of the cited study.^35^ Studies assessing the appetite-related responses during and after single bouts of continuous aerobic exercise indicate that subjective feelings of appetite are transiently suppressed during exercise in people with obesity; but energy intake is minimally affected.^36,37^ Regarding the chronic effects of aerobic exercise on appetite parameters, the results are inconsistent—however, a small increase in hunger at fasting state with a subsequent increase in satiety post-meal and without significant increase on energy intake has been found in a recent systematic review and meta-analysis.^36,38^

Increased appetite likely plays a quantitatively greater role on weight regain than the decreased energy expenditure because the feedback circuits controlling long-term energy intake have greater overall strength compared with the feedback circuits controlling energy expenditure.^30,32^ So, if we consider obesity as a disease of dysregulated appetite where the increased hunger and/or the reduced satiety are the main symptoms,^39^ then lifestyle interventions which usually result in increase of appetite may not effectively treat the symptoms of the underlying disease and this will lead in the majority of the cases to weight regain long-term despite the successful initial WL. However, WL maintenance can occur in a smaller number of people who despite WL with lifestyle interventions do not experience increased appetite, suggesting that lifestyle interventions can also be considered as effective obesity treatments in a small number of patients and that there is individual variability in response to different lifestyle interventions regarding appetite signals and weight regain.^31,40^

Anti-obesity medications can also effectively treat obesity by counteracting the increased drive to eat and the impaired satiation associated with WL by lifestyle changes and could help with further WL and weight maintenance.^41–43^ However, as with most management techniques for chronic diseases, obesity relapses if the treatment is stopped.^44^

### 2.2 The example of bariatric surgery

Bariatric surgery is a collective term for surgical treatment of obesity. It is so far the most successful existing approach for safe and effective obesity treatment and results in sustained WL while at the same time reduces appetite.^11,45^ In the Swedish obese subjects (SOS) study, bariatric surgery was able to induce and maintain ≥15% mean WL over 20 years follow-up (compared with 1% WL at the usual care group).^11^ This amount of sustained WL was associated with improvement in QoL and obesity-related complications, reduction by 53% in fatal and 33% in total cardiovascular events, and reduction by 29% in mortality compared with the usual care group,^11,12,46,47^ providing a WL threshold associated with multiple clinically important benefits. Moreover, bariatric surgery in SOS study reduced the risk for new onset heart failure by 35% and for new diagnosis of atrial fibrillation by 29% compared with the usual care group.^48,49^ The benefits of bariatric surgery on reducing cardiovascular events, cardiovascular death, all-cause mortality and new onset heart failure compared with the non-surgical management are consistent across multiple observational, matched-cohort studies, especially in people with obesity and T2D.^50,51^

The most commonly performed bariatric procedures in the SOS study were gastric band and vertical banded gastroplasty with fewer patients undergoing Roux-en-Y gastric bypass (RYGB).^11^ Today, the two most commonly performed bariatric procedures (≈85% of bariatric procedures worldwide) are sleeve gastrectomy (SG) and RYGB.^52^ RYGB results in 30% WL over the first post-operative year and a sustained 25–27% WL long-term.^11,53^ SG leads to 25% WL at the first post-operative years and around 20% WL long-term.^53^ WL and WL maintenance after bariatric surgery are achieved as a consequence of voluntary reduced food intake due to reduced appetite (*Figure 1*) rather than through restriction, malabsoprtion or increased energy expenditure.^54^ One of the potential mediators for the reduced appetite and food intake after bariatric surgery is the changes in the peripheral signals of body weight regulation (how the gut communicates with the brain) through alteration of gut anatomy. More specifically, both RYGB and SG substantially increase the secretion of multiple satiety gut hormones after food intake, including GLP-1 and PYY and when the action of these hormones was blocked after RYGB, the food intake increased by 20%,^55^ supporting a role of these gut hormones in early post-prandial satiety.

However, not every person with severe and complex obesity wants or is fit enough to undergo surgery, and there is no surgical capacity to operate on every person that qualifies for bariatric surgery. So, pharmacotherapies mimicking some of the post-operative physiological changes after bariatric surgery would be a logical approach to try to achieve similar WL.

### 2.3 Approved pharmacotherapies for obesity over the last decade

Numerous obesity medications targeting appetite and reward centres have been tried in the past to support WL but the majority has been withdrawn due to safety concerns.^56–58^ For example, sibutramine was withdrawn due to increased risk of cardiovascular events (myocardial infarction and non-fatal stroke) in people with obesity and pre-existing cardiovascular conditions when rimonabant was withdrawn due to increased risk of psychiatric adverse events including depressed mood disorders, anxiety, and suicide.^57,58^ More recently, lorcaserin was withdrawn from the market because of a signal of increased cancer risk.^59^ Nevertheless, the better understanding over the last years of the peripheral and central signals and mechanisms involved in WL and WL maintenance have contributed to the development of more effective and safe weight-loss medications. Orlistat, which was approved in 1999 for obesity treatment have demonstrated its safety and efficacy in multiple trials, but it has, at best, modest effect (WL 3–5%) as result of reduced absorption of ingested fat and behavioural changes (to avoid steatorrhea).^60^

Over the past decade, several agents that act by reducing hunger or promote satiation have been approved by regulatory authorities worldwide for chronic weight management, including phentermine plus topiramate (approved only in the USA), bupropion plus naltrexone, and the GLP-1 RAs liraglutide 3 mg and semaglutide 2.4 mg.

#### 2.3.1 Phentermine-topiramate

Phentermine-topiramate (PHEN-TPM) is an oral medication approved for obesity treatment in the USA, but not in Europe due to concerns about the medication’s long-term cardiovascular safety. The fixed-dose combination approved in the USA contains PHEN doses from 3.75 to 15 mg and TPM doses from 23 to 92 mg for daily administration.

PHEN is a sympathomimetic amine which acts as an appetite suppressant via the central nervous system.^61^ It is indicated for short-term use in weight management in USA, however, long-term data are not available. TPM is an anticonvulsant indicated for use in the treatment of migraine and epilepsy.^61^ One of the known effects of TPM is also a decrease in appetite.

In people without diabetes, 56 weeks of PHEN-TPM 15/92 extended release (ER) in combination with 500 kcal/day deficit diet resulted in 10.9% WL compared with 1.6% with placebo and 32.3% of participants achieved more than ≥15% WL.^62^ Similar results were reported at the 2-year follow-up of PHEN-TPM 15/92 ER^63^ (*Table 1*).

**Table 1:** Large multi-centre clinical trials for approved obesity pharmacotherapies over last decade and tirzepatide in populations without diabetes (or with majority of participants without diabetes)

	Lifestyle intervention	Comparator	Duration (week)	BMI baseline (drug/comparator)	WL (drug/comparator) ETD vs. comparator	≥5% WL (%) (drug/comparator)	≥ 10% WL (%) (drug/comparator)	≥ 15% WL (%) (drug/comparator)	Comment
**Phentermine/Topiramate ER** **3.75/23, 7.5/46, or 15/92**									
Gadde, 2011^64^ CONQUER (7.5/46 and 15/92)	500 kcal/day deficit diet + advise for PA	vs. placebo	56	36.6/36.7	−7.8% to −9.8%/−1.2% ETD: −6.6% to – 8.6%	62–70/21	37–48/7	NR	15.6% of participants had T2D
Allison, 2012^62^ EQUIP (3.75/23 and 15/92)	500 kcal/d deficit diet + advise for PA	vs. placebo	56	41.9–42.6/42.0	−5.1% to −10.9%/−1.56% ETD: −3.54% to −9.34%	44.9–66.7/17.3	18.8–47.2/7.4	7.3–32.3/3.4	
Garvey 2012^63^ SEQUEL (7.5/46 and 15/92)	500 kcal/d deficit diet + advise for PA	vs. placebo	108	36.1–36.2/36	−9.3% to −10.5%/−1.8% ETD: −7.5% to –9.7%	75.2–79.3/30	50.3–53.9/11.5	24.2–31.9/6.6	
**Naltrexone/Buproprion SR** **16/360 or 32/360**									
Greenway 2010^65^ COR-I (16/360 and 32/360)	500 kcal/d deficit diet + advise for PA	vs. placebo	56	36.1–36.2/36.2	−5.0% to −6.1%/−1.3% ETD: - 3.7% to −4.8%	39–48/16	20–25/7	9–12/2	
Apovian 2013^66^ COR-II (32/360)	500 kcal/d deficit diet + advise for PA	vs. placebo	56	36.2/36.1	−6.4%/−1.2% ETD: −5.2%	50.5/17.1	28.3/5.7	13.5/2.4	Primary outcome was at 28 weeks
Wadden 2011^67^ COR-BMOD (32/360)	IBT-28 group sessions plus calorie deficit diet and advise for PA	vs. placebo	56	36.3/37	−9.3%/−5.1% ETD: −4.2%	66.4/42.5	41.5/20.2	29.1/10.9	
**Liraglutide 3mg**									
Pi-Sunyer 2015^68^ SCALE-Obesity	500 kcal deficit diet + advise for PA	vs. placebo	56	38.3/38.3	−8.0%/−2.6% ETD: −5.4%	63.2/27.1	33.1/10.6	14.4/3.5	
Le Roux 2017^69^ SCALE-pre-diabetes	500 kcal deficit diet + advise for PA	vs. placebo	160	38.8/39.0	−6.1%/−1.9% ETD: −4.3%	49.6/23.7	24.8/9.9	11/3.1	79% reduction at the risk of developing diabetes in people with pre-diabetes
Wadden 2020^70^ SCALE-IBT	IBT-23 brief sessions plus diet 1200–1800 kcal/d + advise for PA	vs. placebo	56	39.3/38.7	−7.5%/−4.0% ETD: −3.4%	61.5/38.8	30.5/19.8	18.1/8.9	
Wadden 2013 ^41^ SCALE −Maintenance	500kcal deficit diet after achieving ≥5% WL with 1200–1400kcal diet	vs. placebo	56	36/35.2	−6.2%/−0.2% ETD: −6.1%	50.5/21.8	26.1/6.3	NR	Weight maintenance study—participants randomized after they have achieved ≥5% weight loss with diet
Blackman 2016^71^ SCALE sleep apnea	500 kcal deficit diet + advise for PA	vs. placebo	32	38.9/39.4	−5.7%/−1.6% ETD: −4.2%	46.3/18.5	23.4/1.7	NR	Apnoea hypopnea Index (events/hour) reduced by 6.1 events/hour
Kelly 2020^72^ Liraglutide for Adolescents with Obesity	Individualized counselling on healthy nutrition + advise for PA for 60 min/d	vs. placebo	56	35.3/35.8	−2.65%/ +2.37% ETD: −5.01%	43.3/18.7	26.1/8.1	NR	Participants were adolescents 12 to <18 years old
**Semaglutide 2.4mg** ^a^									
Wilding, 2021^15^ STEP-1	500 kcal/day deficit diet + advise for PA	vs. placebo	68	37.8/38.0	−14.9%/−2.4% ETD: −12.4%	86.4/31.5	69.1/12	50.5/4.9	
Wadden 2013^73^ STEP-3	Low-calorie diet (1000–1200 kcal) for 8 weeks and IBT (30 counselling sessions)	vs. placebo	68	38.1/37.8	−16%/−5.7% ETD: −10.3%	86.6/47.6	75.3/27	55.8/13.2	
Rubino, 2021^74^ STEP-4	500 kcal/day deficit diet + advise for PA	vs. placebo	0 →68 20→68	38.4 34.5/34.1	−17.4%/−5.0% −7.9%/ +6.9% ETD: −14.8%	88.7/47.6	79/20.4	63.7/9.2	Medication withdrawal study—all participants received Semaglutide 2.4 mg for 20 weeks and then randomized to placebo vs. Semaglutide 2.4 mg for next 48 weeks
Kadowaki 2022^75^ STEP-6	500 kcal/day deficit diet + advise for PA	vs. placebo	68	86.9/90.2	−13.2%/−2.1% ETD: −11.06%	83/21	61/5	41/3	Study in East Asian population (Japan, South Korea) − 25% of participants had T2D
Rubino, 2022^76^ STEP-8	500 kcal/day deficit diet + advise for PA	**vs. liraglutide 3mg**	68	37/37.2	−15.8%/−6.4% ETD: −9.4%	87.2/58.1	70.9/25.6	55.6/12.0	
**Tirzepatide 5–15mg** ^a^									
Jastreboff A, 2022 SURMOUNT-1^77^	500 kcal/day deficit diet and advise for PA	vs. placebo	72	37.4–38.2/38.2	−15.0% to −20.9%/−3.1% ETD: −11.9% to −17.8%	85.1–90.9/34.5	68.5–83.5/18.8	48.0–70.6/8.8	

WL, weight loss; ER, extended release; SR, slow release; ETD, estimated treatment difference; PA, physical activity; IBT, intensive behavioural therapy; T2D, type 2 diabetes.

^a^Data presented as treatment-policy estimand.

In people with T2D, 56 weeks of PHEN-TPM ER 15/92 reduces body weight by 9.6% compared with 2.6% with placebo and improved HbA1c by 1.6% (17.5 mmol/mol) compared with 1.2% (13.1 mmol/mol) with placebo^78^ (*Table 2*). The most commonly reported adverse events are upper respiratory tract infection, constipation, insomnia, paraesthesia, sinusitis, taste change and dry mouth.^62–64^ PHEN-TPM has also a warning of birth defects (cleft lip and palate) in the offspring of pregnant women taking the medication, due to the known teratogenic effect of TPM.

**Table 2:** Large multi-centre clinical trials for approved obesity pharmacotherapies over last decade and tirzepatide in people with type 2 diabetes

	Lifestyle change	Comparator	Background therapy	Duration (week)	BMI baseline (drug/comparator)	Weight loss (drug/comparator) ETD vs. comparator	HbA1c (%) baseline (drug/comparator)	HbA1c (%) change (drug/comparator)	≥ 10% WL (%) (drug/comparator)	≥ 15% WL (%) (drug/comparator)	HbA1c ≤ 7% (drug/comparator)
**Phentermine/Topiramate ER 15/92**											
Garvey 2014^78^ OB-202/DM-230	500 kcal/day deficit diet and advise for PA	vs. placebo	Diet ± oral glucose lowering meds	56	35.5/35.2	−9.6%/−2.6% ETD: −7%	8.8/8.5	−1.6%/−1.2% ETD: −0.4%	37/9	NR	53/40
**Naltrexone/Buproprion SR 32/360**											
Hollander 2013^79^ COR-Diabetes	500 kcal/d deficit diet + advise for PA	vs. placebo	Not on or stable dose glucose-lowering meds	56	36.7/36.3	−5.0%/−1.8% ETD: −3.2%	8.0/8.0	−0.6%/−0.1% ETD: −0.5%	26.3/8.0		44.1/26.3
**Liraglutide 3mg**											
Davies 2015^80^ SCALE Diabetes	500 kcal/d deficit diet + advise for PA	vs. placebo **vs. liraglutide 1.8 mg**	diet + exercise or ≤ 3 oral glucose-lowering meds	56	37.1/37.4 37.1/37.4	−6.0%/−2.0% ETD: −4% −6.0%/−4.7% ETD: −1.3%	7.9/7.9 7.9/8.0	−1.3/−0.3 ETD: −0.93 −1.3/−1.1 ETD: −0.2	25.2/6.7 25.2/15.9	NR	69.2/27.2 69.2/66.7
Garvey 2020^81^ SCALE-Insulin	IBT-23 sessions	vs. placebo	Basal insulin + ≤ 2 oral glucose-lowering meds	56	35.9/35.3	−5.8%/−1.5% ETD: −4.3%	7.9/8.0	−1.1/−0.6 ETD: −0.5	22.8/6.6	NR	NR
**Semaglutide 2.4 mg** ^a^											
Davies 2021^82^ STEP-2	500 kcal/d deficit diet + advise for PA	vs. placebo **vs. semaglutide 1 mg**	diet + exercise or ≤ 3 oral glucose-lowering meds	68	35.9/35.9 35.9/35.3	−9.64%/−3.42% ETD: −6.21% − 9.64%/−6.99% ETD: −2.65%	8.1/8.1 8.1/8.1	−1.6/−0.4 ETD: −1.2 −1.6/−1.5 ETD: −0.2	45.6/8.2 45.6/28.7	25.8/3.2 25.8/13.7	78.5/26.5 78.5/72.3
**Tirzepatide 5–15 mg**											
Rosenstock 2021^14^ SURPASS-1^a^	No new diet or exercise programme	vs. placebo	Diet and exercise	40	31.5– 32.2/31.7	−6.3 to −7.8 kg/−1.0 kg ETD: −5.3 kg to −6.8 kg	7.80–7.97/8.05	−1.69 to −1.75/−0.09 ETD: −1.6 to −1.66	27 to 38/0	12 to 23/0	78–85/23
Frias 2021^83^ SURPASS-2^a^	Not described	**vs. semaglutide 1mg**	Metformin ≥ 1500 mg/d	40	33.8– 34.5/34.2	−7.6 to −11.2 kg/−5.7 kg ETD: −1.9 to −5.5 kg	8.26–8.32/8.25	−2.01 to −2.3/−1.86 ETD: −0.15 to −0.45	34–57/24	15 to 36/8	82 to 86/79
Ludvik 2021^84^ SURPASS-3^a^	Not described	**vs. insulin degludec**	Metfromin ± SGLT-2i	52	33.4–33.7/33.4	−7 to −11.3 kg/ +1.9 kg ETD: −8.9 to −13.2 kg	8.17–8.21/8.12	−1.85 to −2.14/−1.25 ETD: −0.60 to −0.89	35–58/3	12 to 35/0	79–83/58
Del Prato 2021 ^85^ SURPASS-4^a^	Not described	**vs. insulin glargine**	≤ 3 oral glucose –lowering meds	52	32.5–32.8/32.5	−6.4 to −10.6 kg/ +1.7 kg ETD: −8.1 to −12.3kg	8.52–8.60/8.51	−2.11 to −2.41/−1.39 ETD: −0.72 to −1.02	32–59/2	13–33/ < 1	75–85/49
Dahl 2021^86^ SURPASS-5^a^	Not described	vs. placebo	Insulin glargine ± metformin	40	33.4–33.6/33.2	−5.4 to −8.8 kg/ +1.6 kg ETD: −7.1 to −10.5 kg	8.23–8.36/8.37	−2.11 to −2.40/−0.86 ETD: −1.24 to −1.53	21–42/1	7–24/0	85–90/35
Inagaki N^87^ SURPASS J-mono (Japanese population)	Not described	**vs. dulaglutide 0.75mg**	Diet and exercise	52	28–28.6/27.8	−5.4 to −9.4 kg/−0.4kg^a^ ETD: −4.9 to −8.9 kg	8.2/8.2	−2.24 to −2.57/−1.27^a^ ETD: −1.1 to −1.5	34–67/3^b^	16–45/ < 1^b^	94–99/67^b^
Kadowaki T^88^ SURPASS J-combo^b^ (Japanese population)	Not described	No comparator	1 oral glucose-lowering med	52	27.6–28.4	−3.8 to −10.3 kg^b^	8.5–8.6	−2.5 to −3^b^	20–64^b^	7–41^b^	93–98^b^

WL, weight loss; ER, extended release; SR: slow release; ETD, estimated treatment difference; PA, physical activity; IBT, intensive behavioural therapy; T2D, type 2 diabetes; SGLT-2i: sodium-glucose co-transporters inhibitor.

^a^Data presented as treatment-policy estimand.

^b^Data presented as efficacy estimand, as there is no available data for treatment-policy estimand.

The improvement in weight with PHEN/TPM was associated with improvements in QoL^89^ and cardiometabolic risk factors.^62–64^ A retrospective analysis of PHEN used concurrently with TPM, either separately or in fixed-dose combination showed a trend for a lower rate of major adverse cardiovascular events (MACE) and other cardiovascular outcomes among those receiving PHEN/TPM (including the fixed-dose combination) than among the unexposed cohort (HR: 0.24; 95% CI, 0.03 to 1.70 for fixed-dose PHEN-TPM).^90^ However, the cardiovascular safety of this treatment requires evaluation in an adequately powered outcome trial.

#### 2.3.2 Naltrexone-buproprion

Naltrexone is an opioid receptor antagonist that is approved for the treatment of alcohol and opioid dependence.^91^ Bupropion is a dopamine and norepinephrine reuptake inhibitor that was first approved for the treatment of depression and later for smoking cessation.^91^ The exact mechanism whereby the naltrexone/bupropion (NB) combination leads to WL is not fully understood, but it is likely that it promotes satiety, reduces food intake and may enhance energy expenditure through actions at the hypothalamus and mesolimbic dopamine circuit. Participants in clinical trials who responded to NB as an obesity treatment reported feeling less hungry and more full compared with those receiving placebo and they found it easier to control their food cravings.^65^

In people without diabetes, NB 32/360 together with a 500 kcal deficit diet resulted in 6.3% WL at 52 weeks compared with 0.9% in the placebo group.^65^ In COR-I and COR-II studies, 25–28.3% of those on NB 32/360 achieved ≥10% WL.^65,66^ Additionally, NB 32/360 in combination with an IBT program and diet resulted in 9.3% WL vs. 5.1% with placebo.^67^ In people with obesity and T2D, NB 32/360 resulted in 5% mean WL at 56 weeks compared with 1.8% with placebo.^79^ 26.3% of participants receiving NB 32/360 achieved ≥10% WL and HbA1c was reduced by 0.6% compared with 0.1% in those receiving placebo.^79^

The most common adverse events with NB 32/360 are nausea, headache, constipation, dry mouth, anxiety, dizziness, hypertension, and vomiting.^65,79^ The NB is contraindicated in people with epilepsy as buproprion is associated with dose-related risk of seizures.

Physical function and self-esteem improved with NB 32/360 compared with placebo.^92,93^ The WL achieved with NB 32/360 was also associated with improvements in some cardiometabolic risk factors such as HDL and hepatic insulin resistance markers, however the systolic blood pressure (SBP) and diastolic blood pressure improved more in the placebo group compared with the NB 32/360.^66^

A clinical trial on cardiovascular outcomes for NB was terminated early due to early release of the interim analysis performed after 50% of planned events (HR 0.88; adjusted 99.7% CI 0.57–1.34 compared with placebo).^94^ So, the cardiovascular safety of NB remains uncertain and will require further revaluation in adequately powered outcome trials.

#### 2.3.3 Currently approved pharmacotherapies for obesity based on gut hormones

##### 2.3.3.1 GLP-1 receptor agonists

GLP-1 is an incretin hormone secreted predominantly from L cells located in the small intestine in response to food intake.^95^ In addition to the glucose-lowering actions such as stimulation of glucose-induced insulin secretion, delay in gastric emptying and inhibition of glucagon secretion, exogenous GLP-1 infusion in humans resulted recurrently in reduced calorie intake, reduced appetite and effects on the reward system without direct changes in energy expenditure.^96–98^

GLP-1 RAs have been developed initially for the treatment of T2D, however due to their efficacy in inducing WL and reducing appetite, they have been repurposed in higher doses as treatments for obesity. Exogenous GLP-1 and GLP-1 RAs may predominantly access the brain via the leaks in the blood–brain barrier, where the underlying neuronal tissue shows a dense expression of GLP-1 receptors. In animal experiments, the effect of GLP-1 RAs on food intake is entirely dependent on central nervous system mechanisms,^99^ and in humans this is supported by imaging experiments.^100,101^

##### 2.3.3.2 Liraglutide 3 mg

In 2014, liraglutide 3 mg once daily became the first GLP-1 RA to be approved for the treatment of adults with obesity and in 2021 it was also approved for adolescents ≥12 years old.^72^ Liraglutide 3 mg is a long-acting GLP-1 RA and mechanistic studies have demonstrated that it increases post-prandial satiety and fullness, reduces hunger and prospective food consumption and decreases ad libitum food intake at lunch by ≈16% (136 kcal).^43^ Moreover, the mean 24 h energy expenditure was reduced by ≈5% (139 kcal), which was mainly explained by the decrease in food intake and body weight (*Figure 1*).^43^

Liraglutide 3 mg in combination with a 500 kcal/day deficit diet resulted in 6.1–8% WL in adults without diabetes (*Table 1*).^68,70^ For people with T2D, liraglutide 3 mg resulted in 5.8%—6% WL compared with 1.5–2% WL with placebo (*Table 2*).^80,81^ HbA1c reduced by 1.6% with liraglutide 3 mg and 0.4% with placebo.^80,81^

Liraglutide 3 mg is also effective for weight maintenance after initial WL through diet intervention. In SCALE-Maintenance study, after a 5% WL obtained via a diet over 4–12 weeks, liraglutide 3 mg resulted in further 6.1% WL at 56 weeks compared with placebo (6.2 vs. 0.2%).^41^

The most common side effects include mild to moderate gastrointestinal problems such as nausea, diarrhoea, constipation and (more rarely) vomiting which occurred primarily within the first 4–8 weeks after initiation of liraglutide treatment.^68,80^ Pancreatitis, a rare side effect of GLP-1 RA was reported in 0.7% of participants at liraglutide 3 mg arm [0.3 events per 100 person-years of observation (PYO)] at the 3-year follow-up of SCALE-Prediabetes trial compared with 0.3% of participants at placebo arm (0.1 events per 100 PYO) and the vast majority of the cases were graded as mild.^69^

The QoL and especially the physical function improved more with use of liraglutide 3 mg compared with placebo, with people achieving ≥15% WL having the most benefit.^102^ In addition, WL with liraglutide 3 mg improves also multiple other obesity-related complications, including reduction in T2D incidence by 79% in people with pre-diabetes after 3 years of treatment compared with placebo^69^ and reduction in apnoea-hypopnea index in people with sleep apnoea (however, without change in requirement of continuous positive airway pressure support).^71^

Liraglutide 1.8 mg (dose approved for T2D treatment) was shown to reduce cardiovascular events in patients with T2D and established cardiovascular disease (LEADER trial) followed for up to 5 years.^103^ On the other hand, in a 24-week clinical trial in people with chronic heart failure (left ventricular ejection fraction ≤45%, *n* = 241) with and without diabetes, liraglutide 1.8 mg once daily did not improve the left ventricular systolic function compared with placebo and raised some concerns regarding cardiac safety of the medication at this population.^104^ However, a subanalysis of LEADER trial [including 1667 people with T2D and heart failure at baseline, New York Heart Association (NYHA) functional Class I–III] found that the beneficial effects of liraglutide 1.8 mg vs. placebo on major cardiovascular events and mortality were consistent in people with and without heart failure and there was no difference between the two groups in hospitalization for heart failure, suggesting that liraglutide 1.8mg use is safe for people with T2D and heart failure (NYHA Class I–III).^105^

Liraglutide 3 mg (dose approved for obesity treatment) has been shown to improve multiple cardiometabolic risk factors, but studies to evaluate cardiovascular outcomes in people with obesity have not been done. An analysis of data from SCALE programme demonstrated that liraglutide 3 mg use was not associated with excess cardiovascular risk compared with comparators (HR 0.42, 95% CI 0.17–1.08), however the confidence interval was wide.^106^

##### 2.3.3.3 Semaglutide 2.4 mg

Semaglutide 2.4 mg once weekly is a new long-acting GLP-1 RA which was approved in 2021 for treatment of obesity in the USA and Europe. Semaglutide 2.4 mg reduces energy intake in people with obesity during an ad libitum lunch by 35% (−224 kcal vs. placebo) through appetite reduction.^42^ People received semaglutide reported reduced hunger, increase in fullness and satiety, better control of eating and fewer and weakened food cravings.^42^ Moreover, the rate of gastric emptying was reduced as well as the prospective food consumption for participants receiving semaglutide 2.4 mg.^42^

In clinical trials, 56 weeks of semaglutide 2.4 mg in combination with a 500 kcal/day deficit diet resulted in 14.9% WL vs. 2.4% with placebo in people without diabetes.^15^ 50.5% of those receiving semaglutide 2.4 mg achieved ≥15% WL and 32% achieved ≥20% WL compared with 4.9 and 1.7%, respectively at the placebo group (*Table 1*).^15^ In direct comparison with liraglutide 3 mg, semaglutide 2.4 mg results in more than double WL (*Table 1*, STEP-8).^76^ Additionally, 68 weeks of semaglutide 2.4 mg in combination with IBT and a low-calorie diet during the first 8 weeks resulted in 16% WL vs. 5.7% in the placebo group.^73^

In people with T2D, WL with semaglutide 2.4 mg once weekly was 9.6 vs. 7% with semaglutide 1 mg once weekly (dose approved for T2D treatment) and 3.4% with placebo (*Table 2*).^82^ HbA1c was reduced by 1.6% at 68 weeks with semaglutide 2.4 mg compared with 1.5% with semaglutide 1 mg and 0.4% with placebo (*Table 2*).^82^

Why semaglutide and liraglutide are less effective in people with T2D compared with populations without diabetes is not fully understood, but the different population demographics in the T2D clinical trials (higher percentage of men), the use of background glucose-lowering medications that can contribute to weight gain (such as sulphonylureas) and the reduction of glycosuria due to improvement of glycaemic control with GLP-1 RAs may contribute to these results.^107^

WL with semaglutide was associated with improvements in QoL and participants in the semaglutide group were more likely to have clinically meaningful within-person improvements in physical function than with placebo.^15^ Mild to moderate gastrointestinal problems such as nausea, vomiting and diarrhoea were the most common side effects of semaglutide.^15,108^ Adverse events leading to discontinuation of semaglutide 2.4 mg ranged between 3 and 7% in STEP programme.^15,73–76,82^ Acute pancreatitis was reported in 0.2% of participants at the STEP-1 trial (0.2 events per 100 PYO).^15^

Despite the improvement in multiple cardiovascular risk factors with semaglutide 2.4 mg, the cardiovascular safety profile has not yet been confirmed in people with obesity. A large clinical trial (SELECT, NCT03574597) is currently taking place to assess the effect of semaglutide on major cardiovascular outcomes in people with obesity and established cardiovascular disease. However, in SUSTAIN 6 study, semaglutide 1 mg in people with T2D and established cardiovascular disease resulted in reduction of MACE by 26% compared with placebo (mainly due to reduction in stroke incidence), providing some reassurance.^109^ In SUSTAIN-6, there was no effect of semaglutide 1 mg compared with placebo on hospitalization for heart failure,^109^ but the impact of semaglutide 2.4 mg in people with heart failure with preserved ejection fraction and obesity will be assessed at the ongoing STEP-HFpEF (NCT04788511) and STEP-HFpEF DM (NCT04916470) trials.

Finally, oral semaglutide—an approved treatment for T2D at the doses of 7 and 14 mg once daily—is currently undergoing phase 3 trials as treatment for obesity at the dose of 50 mg once daily. In a phase 2, dose-finding trial, 40 mg of oral semaglutide in people with T2D resulted in 6.9% WL compared with 6.4% with injectable semaglutide 1 mg and 1.2% with placebo.^110^

#### 2.3.4 Summary of approved pharmacotherapies for obesity management

Until 2021, the approved pharmacotherapies for chronic weight management (PHEN/TPM, buproprion/naltrexone, liraglutide 3 mg, orlistat) could result in 5–10% mean WL and WL maintenance when combined with moderate intensity lifestyle interventions. In 2021, Semaglutide 2.4 mg once weekly became the first approved obesity pharmacotherapy which leads to ≈15% WL in people without diabetes when combined with moderate intensity lifestyle interventions, which almost doubles the effectiveness on WL over previous obesity pharmacotherapies with a good tolerability and safety profile in clinical trials.

## 3. Emerging treatments for obesity

### 3.1 Gut hormones-dual agonists and triple agonists

**Figure 2:**
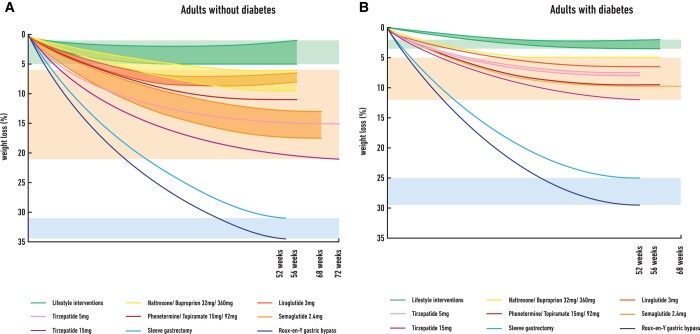
The efficacy gap in weight loss between lifestyle interventions, approved pharmacotherapies for obesity over last decade (plus tirzepatide) and bariatric surgery in adults without type 2 diabetes (*Figure 2A*) and in adults with type 2 diabetes (*Figure 2B*). Data in *Figure 2A* are based on published clinical trials used (*A*) lifestyle interventions (500 kcal deficit diet plus advise for physical activity or intensive behavioural therapy) plus placebo (green background), ^15,62,65–68,70^ (*B*) liraglutide 3 mg,^68,70^ naltrexone/buproprion 32/360,^65–67^ phentermine/topiramate 15/92,^62^ semaglutide 2.4 mg,^15,74–76^ and tirzepatide 5 and 15 mg^77^ plus lifestyle interventions (approved obesity pharmacotherapies plus tirzepatide, orange background) or c) sleeve gastrectomy^112^ and Roux-en-Y gastric bypass^112^ (bariatric surgery, blue background) in adults without type 2 diabetes. Data in *Figure 2B* are based on published clinical trials used a) lifestyle interventions (500 kcal deficit diet plus advise for physical activity or intensive behavioural therapy) plus placebo (green background),^78–80,82^ (*B*) liraglutide 3 mg,^80,81^ naltrexone/buproprion 32/360,^79^ phentermine/topiramate 15/92,^78^ semaglutide 2.4 mg^82^ plus lifestyle interventions, and tirzepatide 5 mg and 15mg^84,85,87^ (approved obesity pharmacotherapies plus tirzepatide, orange background) or c) sleeve gastrectomy^113^ and Roux-en-Y gastric bypass^113^ (bariatric surgery, blue background) in adults with type 2 diabetes.

Although GLP-1 RAs and especially semaglutide are effective treatments for obesity and T2D, there is still significant efficacy gap regarding WL between the currently available pharmacotherapies and bariatric surgery (*Figures* *2A* and *B*). Based on the example of bariatric surgery where multiple gut hormones are increased post-operatively, the combination of GLP-1 with other gut hormones such as glucose-dependent insulinotropic peptide (GIP), glucagon, amylin, and PYY may complement and enhance further the GLP-1 effect leading to additional WL, increased energy expenditure and improved metabolic outcomes.^111^

Tirzepatide is the first dual co-agonist (acting on GLP-1/GIP receptors) which has been approved for treatment of T2D based on the findings of the clinical trials from the SURPASS programme. Moreover, tirzepatide is currently undergoing an extensive programme of phase 3 clinical trials as treatment for obesity (SURMOUNT). The recently published SURMOUNT-1 trial randomized 2539 participants with obesity (without diabetes) to three different doses of tirzepatide (5, 10, and 15 mg, *n* = 1896) or placebo (*n* = 643) with follow-up 72 weeks and showed that tirzepatide 5–15 mg results in 15–20.9% WL when combined with moderate intensity lifestyle interventions compared with 3.1% WL with placebo.^77^

Other dual co-agonists acting on GLP-1/amylin receptors and GLP-1/glucagon receptors are also being assessed in early phase clinical trials as potential treatments for obesity and metabolic complications.^114,115^ Moreover, triple agonists, for example, GLP-1/GIP/glucagon agonists are being explored as potential treatments, although data from clinical trials are limited and in early stage.

### 3.2 GLP-1/GIP combinations for people with obesity and T2D

GIP is a 42-amino acid peptide secreted by endocrine K cells in the duodenum and jejunum in response to nutrient ingestion and is an incretin hormone. In people without diabetes, GIP stimulates insulin secretion but does not change glucagon release during hyperglycaemia, whereas it increases glucagon release without affecting insulin secretion during hypoglycaemia.^116,117^ In the context of T2D, the ability of GIP to stimulate insulin secretion and to ameliorate glycaemia is impaired.^118^

Regarding the effect of GIP on appetite, it has only been assessed in a few acute studies in humans.^101,119,120^ Unlike GLP-1, exogenous GIP infusion does not seem to reduce appetite and the combination of GLP-1 with GIP in people with and without T2D does not lead to any more significant appetite reduction compared with GLP-1 alone.^101^

On this background, it came as a surprise that the GLP-1/GIP co-agonist, tirzepatide, had glucose-lowering and weight-loss activities that exceeded those of GLP-1 RA comparators in clinical trials.^83,121^ Tirzepatide is a once weekly unimolecular agonist of both GLP-1 and GIP receptors which has a deliberate bias towards GIP over GLP-1 activity (it possesses five-fold increased relative potency at human GIP receptor when compared with GLP-1 receptor).^122^ This has been provided as a potential explanation for the more prominent effects on weight and glucose compared with the more balanced dual agonists targeting GLP-1 and GIP receptors equally (although this is difficult to understand given that GIP alone has no effect on food intake in man). Further research is required to understand the mechanisms mediating the effects of tirzepatide on weight and glycaemia. Compared with GLP-1, however, tirzepatide acts as a biased agonist with little beta-arrestin recruitment and receptor internalization, which may explain the superior activity on target cells.^123^

The phase 3 trials with tirzepatide doses 5–15 mg in people with T2D who are overweight or have obesity (SURPASS programme) have shown marked improvements both on WL and glycaemia, despite that tirzepatide in SURPASS programme was not combined with lifestyle changes (as primary outcome was improvement in glycaemia rather than WL), such as in the SCALE and STEP programmes. In SURPASS-3 study, 52 weeks of tirzepatide 15 mg led to mean WL of 11.3 kg (treatment-policy estimand) compared with 1.9 kg weight gain with insulin degludec and 35% of those on tirzepatide 15 mg were able to achieve ≥15% WL compared with 0% at the insulin degludec group (*Table 2*).^84^ Moreover, HbA1c reduced with tirzepatide 15 mg by 2.14% (treatment-policy estimand) in SURPASS-3.^84^ Similar findings regarding WL and glycaemic improvement after 52 weeks of tirzepatide use were also reported at the SURPASS-4 trial (*Table 2*). ^85^

In direct comparison with semaglutide 1 mg (dose for treatment of T2D), tirzepatide 15 mg led to 5.5 kg more WL at 40 weeks and 36% of people achieved ≥15% WL compared with 8% with semaglutide 1 mg (SURPASS-2).^83^ Tirzepatide 15 mg resulted also in an HbA1c reduction of 0.45% more compared with semaglutide 1 mg (*Table 2*).^83^

The safety and efficacy of tirzepatide as treatment for obesity in people without diabetes has been assessed at the recently published SURMOUNT-1 clinical trial. The study found that tirzepatide 5–15 mg together with a moderate intensity lifestyle intervention for 72 weeks resulted in 30–57% of participants achieving ≥20% WL and 15–36% ≥ 25% WL compared with 3 and 1.5% with placebo, respectively.^77^ The physical function was improved more with tirzepatide compared with placebo at the SURMOUNT-1 study and the mean reduction in total body fat mass was 33.9% with tirzepatide compared with 8.2% with placebo.^77^ Between participants with pre-diabetes, 95.3% had reverted to normoglycaemia in the tirzepatide group at 72 weeks, when compared with 61.9% of participants in the placebo group.^77^

Nausea, diarrhea, vomiting, and constipation were the most commonly reported side effects both in SURPASS programme and in SURMOUNT-1 study. Most of them were minor to moderate in severity and temporary. Tirzepatide did not increase the risk of hypoglycaemia in SURPASS programme unless it was combined with insulin or sulfonylureas.^14,85,86^ Decreased appetite was also a commonly reported side effect. Adverse events leading to discontinuation of medication ranged from 3–11% with tirzepatide 5 mg to 7–11% with tirzepatide 15 mg at SURPASS programme and between 4.3 and 7.1% at SURMOUNT-1.^14,77,83–86^ In SURMOUNT-1, the incidence of pancreatitis was low and similar between tirzepatide and placebo group (0.2% in each group), when cholecystitis was reported more frequently with tirzepatide compared with placebo (the overall incidence was still low, < 0.6%), possibly due to the considerable weight reduction with the medication.^77^

Cardiometabolic risk factors (SBP, total cholesterol, HDL, and LDL) are all improved with tirzepatide in people with and without T2D.^14,77,85^ In SURPASS-4 study, people with T2D and increased cardiovascular risk were randomized to tirzepatide 5–15 mg (997 participants) or insulin glargine (1005 participants) for at least 52 weeks, with treatment continued for a maximum of 104 weeks aiming to provide some initial evidence on cardiovascular safety of tirzepatide.^85^ An adjudicated 4-point MACE (cardiovascular death, myocardial infarction, stroke, hospitalization for unstable angina) occurred in 109 participants and was not increased on tirzepatide compared with glargine (HR 0.74, 95% CI 0.51–1.08).^85^ Additionally, a pre-specified cardiovascular meta-analysis including all seven randomized controlled trials from the SURPASS programme compared the time to first occurrence of a 4-point MACE (cardiovascular death, myocardial infarction, stroke, and hospitalized unstable angina) between pooled tirzepatide groups and control groups.^124^ Overall, 142 participants (109 from the SURPASS-4 study) had at least one MACE-4 event. The hazard ratios comparing tirzepatide vs. controls were 0.80 (95% CI, 0.57–1.11) for MACE-4; 0.90 (95% CI, 0.50–1.61) for cardiovascular death; and 0.80 (95% CI, 0.51–1.25) for all-cause death.^124^ These results suggest that that there is no excess cardiovascular risk with tirzepatide, however the definite impact of tirzepatide on cardiovascular disease in people with T2D will be addressed in the ongoing SURPASS-CVOT study (NCT04255433). The impact of tirzepatide in people with heart failure with preserved ejection fraction and obesity will also be assessed at the SUMMIT trial (NCT04847557).

Overall, the GLP-1/GIP co-agonist tirzepatide, is the first approved treatment for T2D which at the higher dose (15 mg) consistently leads to >10% mean WL in studies with at least 52 weeks follow-up (*Table 2*), despite that at the SURPASS programme there was no additional lifestyle intervention. Moreover, in people with obesity (without diabetes) tirzepatide 10 and 15 mg when combined with moderate intensity lifestyle interventions can lead to ≈20% mean WL with good tolerability and similar side effect profile to that of GLP-1 RAs.

## 4. A new era in obesity pharmacotherapy

**Figure 3:**
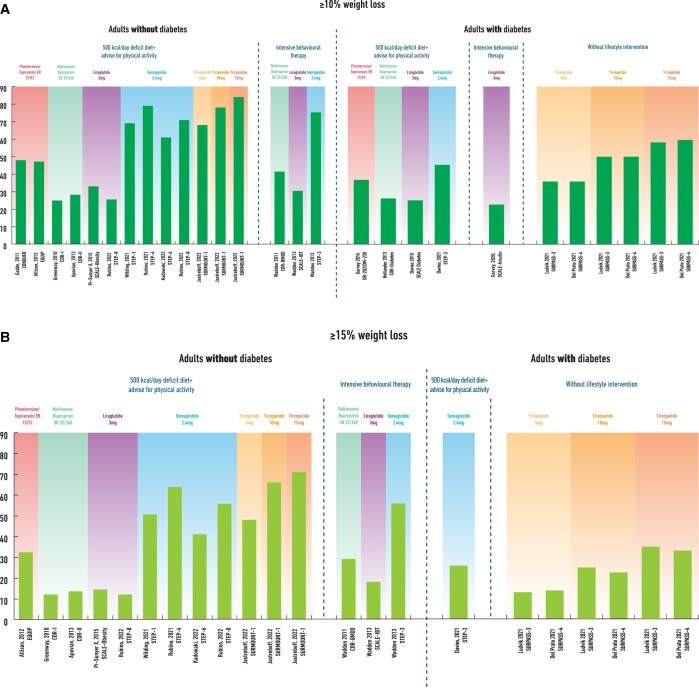
Categorical weight loss (≥10% in *Figure 3A*, ≥ 15% in *Figure 3B*) in large clinical trials with the approved obesity pharmacotherapies over the last decade (plus tirzepatide) in people with and without type 2 diabetes (studies with follow-up 52–72 weeks).

The WL achieved with semaglutide 2.4 mg in people with obesity and the dual agonist tirzepatide in people with T2D and/or obesity suggests that we are entering a new era in obesity pharmacotherapy where ≥15% WL and maintenance is feasible (*Figure* *3A* and *B*).

These obesity treatments will be presented to patients only if the healthcare providers understand that obesity is a chronic disease which is difficult to treat with lifestyle changes alone and the management usually requires a multimodal treatment strategy including pharmacotherapy.^107^ Both clinicians and public or private payers need to understand that sustained WL requires lifelong treatment of obesity as otherwise weight regain will occur when treatment is discontinued.^44^ There are however important research priorities over the next years to allow the new obesity pharmacotherapies to become more widely acceptable and available.

### 4.1 The cardiovascular safety of new pharmacotherapies for obesity

In the past, multiple WL medications have been withdrawn due to safety concerns.^56–58^ Today, good quality evidence is lacking regarding the cardiovascular benefits of the approved pharmacotherapies for obesity as discussed before, although the results of the cardiovascular outcome trials conducted in people with T2D are likely to also apply to people with obesity. Currently, the SELECT study is taking place and will assess the effect of semaglutide 2.4 mg on cardiovascular outcomes in people with obesity and established cardiovascular disease but without diabetes. Moreover, SURPASS-CVOT study will compare dulaglutide vs. tirzepatide on cardiovascular outcomes in people with T2D and established cardiovascular disease and will provide evidence on whether the dual GLP-1/GIP co-agonist is as safe as GLP-1 RAs. Future studies will be needed to establish the safety and benefits of the new medications, including the new combinations of gut hormones.

### 4.2 How we can best combine the new pharmacotherapies with lifestyle interventions to achieve weight loss targets and healthy weight maintenance?

The majority of the clinical trials combine pharmacotherapies for obesity with moderate intensity lifestyle changes. However, intensive lifestyle interventions including low-calorie diets and structured exercise may be used to support WL in clinical practice. How to best combine an intensive lifestyle intervention with pharmacotherapies for obesity treatment requires further investigation.

A single-centre clinical trial assessed the efficacy in weight maintenance of liraglutide 3 mg and/or a 52-week structured exercise programme vs. placebo and continuing with usual physical activity, in people who achieved at least 5% WL through an 8-week low-calorie diet (‘responders’ to low-calorie diet).^21^ The combination of liraglutide 3 mg with a structured exercise programme (*n* = 49) resulted in 15.7% mean total WL at 60 weeks after initiation of the low-calorie diet with 49% of participants achieving ≥15% WL and 32% achieving ≥20% WL.^21^ In participants receiving placebo and continuing with usual activity (*n* = 49) after at least 5% WL with the 8-week low-calorie diet, mean WL at 60 weeks was 6.2% and only 10% of participants were able to achieve ≥15% WL.^21^ Importantly, the addition of the exercise programme also caused a greater loss of fat mass, preservation of lean mass, improved cardiorespiratory fitness and overall improvement in cardiometabolic risk factors compared with the WL obtained with liraglutide 3 mg alone.^21^ The WL achieved in this trial (at 60 weeks) with the combination of liraglutide 3 mg plus structured exercise for weight maintenance in people who were ‘responders’ to the initial 8-week low-calorie diet is similar to the WL reported with semaglutide 2.4 mg once weekly at 68 weeks when combined with a 500 kcal deficit diet^15^ (STEP-1 study, *Table 1*) and significantly more than the 8% WL in SCALE-Obesity where liraglutide 3 mg was combined with 500 kcal deficit diet and advise for physical activity (*Table 1*).^68^

On the other hand, in the STEP-3 study where semaglutide 2.4 mg was combined with IBT and an 8-week low-calorie diet,^15,73^ the WL achieved at 68 weeks was similar to the WL reported when Semaglutide 2.4 mg was combined with a 500 kcal/day deficit diet^15^ (16 vs. 14.9%) and less when compared with patients who could tolerate Semaglutide 2.4 mg in the STEP 4 study (16 vs. 17.4%).^74^ These results suggest the possibility that intensive lifestyle treatments may not be able to provide additional WL to effective obesity medications such as semaglutide and tirzepatide. However, lean muscle mass loss remains a challenge, because with effective obesity treatments patients consume small amount of calories and may not be enough space in their diet to ensure adequate protein intake, thus rendering them catabolic and burning muscle mass. The addition of exercise to these effective pharmacological interventions may further improve body composition, physical function, and cardiorespiratory fitness.^21^ Further studies are necessary to help us understand how to best combine the new pharmacotherapies with different lifestyle interventions not only to maximize WL, but also to optimize body composition and health benefits.

Moreover, the sustainability of WL achieved through different combinations of intensive lifestyle and pharmacotherapy needs to be assessed with long-term studies, together with the changes in body composition, appetite, and energy expenditure over time.

### 4.3 The long-term clinical effectiveness and cost-effectiveness of new pharmacotherapies

Similar to treatments for other chronic diseases such as hypertension or diabetes, at the moment that the treatment for obesity stops the disease will relapse and body weight will increase again, as was seen in the STEP 1 extension study.^44^ In this exploratory analysis, a representative subset of participants (*n* = 327) who completed 68 weeks of treatment with semaglutide 2.4 mg in STEP-1 trial and achieved mean WL of 17.3% of their baseline weight, underwent an off-treatment extension (including lifestyle intervention) for an additional year.^44^ One year after withdrawal of semaglutide 2.4 mg and lifestyle intervention, participants regained two-thirds of their prior WL resulting in final total WL of 5.6% from baseline weight.^44^ Cardiometabolic improvements seen with semaglutide treatment were also reverted towards baseline at the end of the off-treatment extension period for most parameters.^44^

Additionally, in the STEP-4 trial, adults who were overweight or had obesity completed a 20-week run-in of weekly treatment with subcutaneous semaglutide, 2.4 mg, with a mean WL of 10.6%, and then were randomized to continued treatment with subcutaneous semaglutide vs. placebo for an additional 48 weeks.^74^ At the end of the trial, people receiving semaglutide 2.4 mg lost a further 7.9% of their body weight whereas those on placebo regained 6.9% of their body weight (almost 70% of the weight lost).^74^

These results suggest that ongoing treatment is required to maintain improvements in weight, although this topic so far has been poorly explored. Perhaps, an initial WL could be maintained long-term with lower doses of anti-obesity medications. For example, 1.2 mg liraglutide was sufficient to maintain WL for a year after an initial 12% WL obtained with a low-calorie diet.^125^

Currently, there is a lack of long-term data on the effectiveness and safety of obesity pharmacotherapies and on the improvement in obesity-related comorbidities (so far the longest follow-up study is for liraglutide 3 mg up to 3 years after initiation of treatment).^69^ The release of the STEP-5 study results (presented but not published yet) demonstrate that 104 weeks of semaglutide 2.4 mg once weekly results in 15.2% WL compared with 2.6% with placebo, with 36.1% of participants in semaglutide group achieving ≥20% WL at the end of the study.^126^

Studies in real-world settings with long-term follow-up on the new obesity pharmacotherapies may also provide detailed information about the cost-effectiveness of these interventions and may facilitate their approval by public and private payers for long-term use. Long-term data will provide additional information on the potential long-term side effects of obesity pharmacotherapy including adverse events due to significant long-term WL. WL through bariatric surgery has been associated with increased risk for fractures^127^ and increased risk of self-harm behaviors^128^—whether these complications will be observed with the new pharmacotherapies need further investigation.

In summary, further evidence on the long-term safety (especially cardiovascular safety), clinical effectiveness and cost-effectiveness of the new pharmacotherapies for obesity in real-world settings will support their wider use and acceptability by healthcare professionals, individuals living with obesity and healthcare systems.

## 5. Other gut hormones in the pipeline as potential future therapeutic candidates

### 5.1 Amylin analogues

Amylin is a pancreatic β-cell hormone that is co-secreted with insulin in response to food intake.^129^ It functions as a satiety signal, acting upon brain regions involved in both homeostatic and hedonic appetite regulation; it also slows the gastric emptying, and thereby suppresses post-prandial glucagon responses to meals.^129^

Cagrilintide is a weekly subcutaneous amylin analogue that is under development as treatment for obesity. In a phase 2 study, people with obesity were randomly assigned to cagrilintide 0.3–4.5 mg, liraglutide 3.0 mg, or placebo for 26 weeks.^114^ Cagrilintide led to dose-dependent weight reductions and greater WL at all doses compared with placebo at 26 weeks.^114^ WL with cagrilintide 4.5 mg (10.8%) was greater than with liraglutide 3.0 mg (9.0%) or placebo (3.0%).^114^ Cagrilintide 4.5 mg resulted in ≥10% WL in 53.5% of participants and ≥15% WL in 18.7% of participants.^114^ Gastrointestinal disorders were the most common adverse events, primarily nausea.^114^

Different doses of cagrilintide were also evaluated in a phase 1b study in combination with semaglutide 2.4 mg. At 20 weeks, cagrilintide 2.4 mg and semaglutide 2.4 mg led to 17.1% WL compared with 9.8% loss with semaglutide 2.4 mg plus placebo.^130^ This increased WL was not accompanied by worsening tolerability, suggesting that the two apparently complementary mechanisms of action may be combined for potential additive WL. Further clinical trials assessing the safety and efficacy of the combination of semaglutide plus cagrilintide as treatment of obesity are expected to take place over next years.

### 5.2 Glucagon agonists

Glucagon is a 29-amino acid peptide that is secreted from pancreatic α-cells in response to low levels of blood glucose or increasing levels of amino acids. It increases blood glucose through stimulation of glycogenolysis in the liver, but it also reduces food intake, increases satiety and possibly energy expenditure.^131^

The concept of GLP-1/glucagon co-agonists includes the concurrent activation of the GLP-1 receptors leading to decreased energy intake and the glucagon receptors causing increased energy expenditure and reduced energy intake. Animal studies with potent GLP-1/glucagon co-agonists were promising, however the results of the clinical studies in humans for the dual GLP-1/glucagon receptor agonist cotadutide, currently under development, were less impressive.^115,132^ In a phase 2b, randomized, double-blind study, adults who were overweight or had obesity with T2D were randomized to receive cotadutide 100 , 200 or 300 μg; placebo; or open-label liraglutide 1.8 mg for 54 weeks.^115^ The body weight reduction with cotatutide 300 μg was 5.02% at week 54 (compared with −0.68% in placebo group and −3.33% with liraglutide 1.8 mg) and 15.5% of people on cotadutide 300 μg achieved ≥10% WL. Cotadutide also significantly lowered HbA_1c_ by 1.03–1.19% at week 54 while reduction with placebo was 0.45 and 1.17% with liraglutide 1.8 mg.^115^ Gastrointestinal disorders, including diarrhoea, nausea, and vomiting, were the most commonly reported adverse events with cotadutide at any tested dose and more patients stopped cotatutide due to side effects compared with placebo or liraglutide 1.8 mg.^115^ However, glucagon is also thought to increase hepatic lipid oxidation and inhibit lipogenesis and ad hoc analysis of this trial with cotatutide demonstrated improvements in hepatic parameters and supports further evaluation of cotadutide in non-alcoholic steatohepatitis.^115^

Another GLP-1/glucagon receptor dual agonist (SAR425899) was recently evaluated in single-ascending dose and multiple-ascending dose phase 1 trials where it was given once a day over 28 days.^133^ At the highest maintenance doses tested, there was a reduction of HbA1c by 0.54–0.59% when given to patients who were overweight or had obesity with T2D, and mean WL of 2.4–5.5 kg over the 28 days.^133^ SAR425899 was generally well tolerated, with treatment-emergent adverse effects of reduced appetite and nausea.^133^

Regarding triple agonists, evidence from experimental studies in animals suggests that the addition of GIP activity into dual GLP-1 and glucagon receptor agonism provides improved WL and glycaemic control while protecting against the diabetogenic risk of chronic glucagon agonism.^133,134^ Importantly, the addition of the GIP component may allow an increased potency of the agonist at the glucagon receptor. However, very limited data is available from clinical trials—a phase 1 clinical study in healthy individuals (lean to overweight) with a new unimolecular GLP-1, GIP, and glucagon receptor triagonist (SAR441255) showed that the triagonist improved glycaemic control during a mixed-meal tolerance test and was well tolerated.^134^ Additionally, a recent abstract presented the results of a 12-week, phase 1 study, where the safety and tolerability of multiple doses of another GLP-1/GIP/glucagon co-agonist (LY3437943) compared with placebo were assessed in 72 people with T2D. The triple co-agonist showed similar safety and tolerability profile to other incretins and led to placebo-adjusted reduction in HbA1C of up to 1.56% and to placebo-adjusted weight reduction of up to 8.96 kg.^135^ Further trials in larger populations of people with T2D and obesity are required to confirm the therapeutic potential of GLP-1/GIP/glucagon receptor triagonists.

### 5.3 PYY analogues

PYY is a peptide hormone that is co-secreted with GLP-1, particularly from distal epithelial L cells in the gut in response to food intake. PYY3–36 is the active form of the peptide and acts as a satiety hormone, suppressing food intake via activation of Y2 receptors in the hypothalamus. Infusion of PYY3–36 in people with obesity causes a 30% reduction in food intake.^136^ A recent phase 1 study investigating a long-acting PYY3–36 analogue demonstrates a reduction of 38–55% in food intake vs. placebo at 30 days after initiation of treatment and WL of 2.87–3.58 kg compared with placebo.^137^

The co-administration of GLP-1 and PYY3-36 in humans also reduced energy intake compared with placebo and more than the sum of the individual infusions, demonstrating a synergistic effect.^138,139^ A long-acting PYY analogue in combination with semaglutide is now being assessed in a phase 2 study as treatment for obesity (NCT04969939).

### 5.4 Summary of potential future obesity treatments based on gut hormones

Except of the approved GLP-1 RAs for weight management and the GLP-1/GIP co-agonist tirzeparide which has completed phase 3 trials as treatment for T2D and/or obesity, multiple other gut hormones such as amylin, glucagon and PYY are being tested in early phase clinical trials as potential treatments for obesity and obesity-related complications, either as monotherapies (amylin, PYY) or in combination with GLP-1 as dual (GLP-1/amylin, GLP-1/PYY, and GLP-1/glucagon) and triple co-agonists (GLP-1/GIP/glucagon). A phase 3 trial assessing the efficacy and safety of the combination of semaglutide 2.4 mg with cagrilintide 2.4 mg once weekly for people with obesity and T2D is expected to start at the last trimester of 2022 (REDEFINE 2, NCT05394519). In the near future, there is a real prospect of the above described gut hormone combinations to deliver improved weight-related outcomes over the currently available treatments for obesity and T2D.

## 6. Obesity treatments not based on gut hormones

It is common practice in chronic diseases such as hypertension or diabetes to target multiple mechanisms to achieve optimal disease management. Similarly, in obesity, despite that research has mainly focused in combinations of gut hormones, new treatments targeting different pathways such as Setmelanotide and Bimagrumab have also been developed.

Setmelanotide is an melanocortin-4 receptor agonist that reduces bodyweight and hunger in individuals with ultra-rare obesity genetic disorders caused by leptin receptor (LEPR) or pro-opiomelanoctin (POMC) deficiency (80% of participants in POMC trial and 45% of participants in LEPR trial achieved at least 10% WL after 1 year of medication use).^140^ Individuals with these genetic variants have severe hunger (hyperphagia) and early-onset severe obesity resulting from disruption at the melanocortin pathway, which plays pivotal part in body weight regulation.^140^ Setmelanotide has now been approved in the USA and Europe for chronic weight management in patients 6 years and older with obesity resulting from POMC, PCSK1, or LEPR deficiency confirmed by genetic testing.

Bimagrumab is a fully human monoclonal antibody that binds the activin type 2 receptors and prevents the actions of natural ligands, including myostatin and activin A, that otherwise negatively regulate skeletal muscle growth.^141,142^ In phase 2 clinical trials, 48 weeks of treatment with Bimagrumab in individuals living with obesity and T2D resulted in WL of 6.5% compared with 0.5% (−0.18 kg) in the placebo group, with a reduction in total body fat mass by 20.5% (−7.49 kg) and an increase in the lean mass by 3.6%, suggesting that Bimagrumab could be a potential treatment for sarcopenic obesity. HbA1c fell by 0.76% compared with a rise by 0.04% with placebo.^141^ Dietary intake based on 24 h recall did not differ from baseline at the end of the study, suggesting that increase in energy expenditure could be a mechanism of action for the WL with this medication.^141^

Overall, Setmelanotide is considered the first approved personalized treatment for obesity (for individuals with confirmed ultra-rare obesity genetic disorders) when the early phase trials with Bimagrumab provide evidence that we may succeed in improving the quality of WL and preserve lean mass in the treatment of obesity.

## 7. Conclusion

WL ≥10% and maintenance is challenging with lifestyle changes alone due to compensatory increases in appetite and reduction in energy expenditure. Bariatric surgery is currently the most effective intervention for sustained WL ≥20% and leads to multiple health benefits, but surgical procedures are difficult to scale to treat the entire population. Over the last decade, a number of medications have been approved for chronic weight management in people with obesity but Semaglutide 2.4 mg once weekly (a new GLP-1 RA) is the first one which leads to ≈15% WL (in people without diabetes). Moreover, the WL achieved in people with T2D and/or obesity at phase 3 clinical trials with the recently approved for T2D management dual agonist tirzepatide suggests that combination of gut hormones may lead to additional WL compared with GLP-1 RAs alone. Judging from the results of clinical trials in individuals with T2D, these treatments may also have beneficial cardiovascular effects. Additional research assessing long-term safety, effectiveness, and cost-effectiveness of these new pharmacotherapies (semaglutide 2.4 mg and tirzepatide) in trials and real-world settings will help us to understand better their position in the weight management algorithms for people with obesity and/or T2D. Furthermore, novel pharmacological interventions combining GLP-1 with other gut hormones are currently under development and may offer in the future the potential to bridge further the efficacy gap between bariatric surgery and the currently available pharmacotherapies.

## Data Availability

Data derived from sources in the public domain. Reference details are provided in full.
